# CXCR4-Targeted Radiopharmaceuticals for the Imaging and Therapy of Malignant Tumors

**DOI:** 10.3390/molecules28124707

**Published:** 2023-06-12

**Authors:** Jingjing Yu, Xu Zhou, Langtao Shen

**Affiliations:** 1 HTA Co., Ltd., Beijing 102413, China; yujingjing@circ.com.cn; 2 Department of Nuclear Technology Application, China Institute of Atomic Energy, Beijing 102413, China; 3 National Isotope Center of Engineering and Technology, China Institute of Atomic Energy, Beijing 102413, China

**Keywords:** CXCR4, chemokine receptor, CXCL12, radiopharmaceuticals, metastatic cancer

## Abstract

C-X-C chemokine receptor type 4 (CXCR4), also known as fusin or CD184, is a 7-transmembrane helix G-protein-coupled receptor that is encoded by the CXCR4 gene. Involved in various physiological processes, CXCR4 could form an interaction with its endogenous partner, chemokine ligand 12 (CXCL12), which is also named SDF-1. In the past several decades, the CXCR4/CXCL12 couple has attracted a large amount of research interest due to its critical functions in the occurrence and development of refractory diseases, such as HIV infection, inflammatory diseases, and metastatic cancer, including breast cancer, gastric cancer, and non-small cell lung cancer. Furthermore, overexpression of CXCR4 in tumor tissues was shown to have a high correlation with tumor aggressiveness and elevated risks of metastasis and recurrence. The pivotal roles of CXCR4 have encouraged an effort around the world to investigate CXCR4-targeted imaging and therapeutics. In this review, we would like to summarize the implementation of CXCR4-targeted radiopharmaceuticals in the field of various kinds of carcinomas. The nomenclature, structure, properties, and functions of chemokines and chemokine receptors are briefly introduced. Radiopharmaceuticals that could target CXCR4 will be described in detail according to their structure, such as pentapeptide-based structures, heptapeptide-based structures, nonapeptide-based structures, etc. To make this review a comprehensive and informative article, we would also like to provide the predictive prospects for the CXCR4-targeted species in future clinical development.

## 1. Introduction

### 1.1. Chemokines

The chemokines (chemotactic cytokines) are a series of small, mostly secreted proteins that consist of about 60 to 90 amino acids (8–10 kDa in mass) with an N terminal and a C terminal. At the N terminals of these proteins, there are two or four cysteine residues. In the biological environment, the primary function of chemokines is to induce cell migration [1].

Up until now, there have been about 50 kinds of chemokines with different combinations of amino acids. These structures could be divided into four groups (CC, CXC, CX3C, and XC chemokines) according to the spacing of the first two cysteine residues at the N terminals. For example, there are two adjacent cysteine residues at the N terminals of CC chemokines; there are two cysteine residues that are separated by one other amino acid in CXC chemokines; there are two cysteine residues that are separated by three other amino acids in CX3C chemokines; there is only one cysteine residue at the N terminal of XC chemokines, and another cysteine residue is lacking [2].

### 1.2. Chemokine Receptors

The chemokine receptors are endogenous partners of chemokines, and they could form interactions with their partners to signal in a series of biological processes. They are 7-transmembrane helix G-protein-coupled receptors. Just as with chemokines, chemokine receptors could also be divided into four subgroups, including CCR, CXCR, CX3CR, and XCR [3]. Table 1 briefly shows the combinations of chemokines and their receptors.

All these chemokine receptors mediate chemokine functions in their target cells. Chemokine receptor activation will lead to protein kinase activation and intracellular Ca^2+^ mobilization. It is worth mentioning that the interaction between chemokines and their receptors is associated with various functions of normal cells, such as cell proliferation, differentiation, activation and polarization of blood cells, regulation of intracellular calcium levels, chemotaxis, and gene transcription [4]. Overall, the function of chemokines is essential for the induction of the migration of immune cells. Furthermore, the overexpression of chemokine receptors in different types of autoimmune diseases, such as rheumatoid arthritis, systemic lupus erythematosus, multiple sclerosis, Alzheimer’s disease, stroke, HIV, and metastatic cells, can be used as excellent candidates to provide comprehensive data for the diagnosis and treatment of diseases in vivo and in vitro.

**Table 1:** Chemokines and their receptors ^1^.

Chemokine Receptor	Chemokine
CCR1	CCL1, CCL3, CCL4, CCL5, CCL8, CCL14, CCL15, CCL16
CCR2	CCL2, CCL7, CCL8, CCL12, CCL13, CCL16
CCR3	CCL5, CCL7, CCL8, CCL11, CCL13, CCL15, CCL23, CCL24, CCL26, CCL28
CCR4	CCL17, CCL22
CCR5	CCL3, CCL4, CCL5, CCL7, CCL8, CCL13, CCL16
CCR6	CCL20, CCL21
CCR7	CCL19, CCL21
CCR8	CCL1, CCL8, CCL18
CCR9	CCL25
CCR10	CCL27, CCL28
CXCR1	CXCL6, CXCL8
CXCR2	CXCL1, CXCL2, CXCL3, CXCL5, CXCL6, CXCL7, CXCL8
CXCR3	CXCL9, CXCL10, CXCL11
CXCR4	CXCL12
CXCR5	CXCL13
CXCR6	CXCL16
CXCR7	CXCL11, CXCL12
CXCR9	CXCL16
CX3CR1	CX3CL1, CCL26
XCR1	XCL1, XCL2

^1^ This table is summarized according to references [5,6,7].

### 1.3. CXCR4

C-X-C chemokine receptor type 4 (CXCR4), also known as fusin or CD184, is a 7-transmembrane helix G-protein-coupled receptor that is encoded by the CXCR4 gene. One surface of CXCR4 is rich in aspartate and glutamate residues, which could bind firmly to transition metals. CXCR4 is an HIV coreceptor that mediates HIV infection [8]. Overexpression of CXCR4 is present in the majority of cancers [9]. This phenomenon is often correlated with an aggressive tumor phenotype, elevated risks of metastasis and recurrence of the primary tumor, and a poor prognosis of the disease.

Involved in various physiological processes, CXCR4 could form an interaction with its endogenous partner, chemokine ligand 12 (CXCL12), which is also named SDF-1 [10]. In the past several decades, the CXCR4/CXCL12 couple has attracted a large amount of research interest due to its critical functions in the occurrence and development of refractory diseases, such as HIV infection, inflammatory diseases, and metastatic cancer, including breast cancer, gastric cancer, and non-small cell lung cancer [11]. The CXCR4-CXCL12 axis could promote angiogenesis and recruit myeloid bone marrow-derived cells to facilitate tumor recurrence and metastasis, thus mediating resistance to conventional as well as targeted therapies. Neutralization of CXCR4/CXCL12 chemotaxis using anti-CXCR4 antibodies, peptide antagonists, or small molecule antagonists could significantly reduce the metastasis.

**Figure 1:**
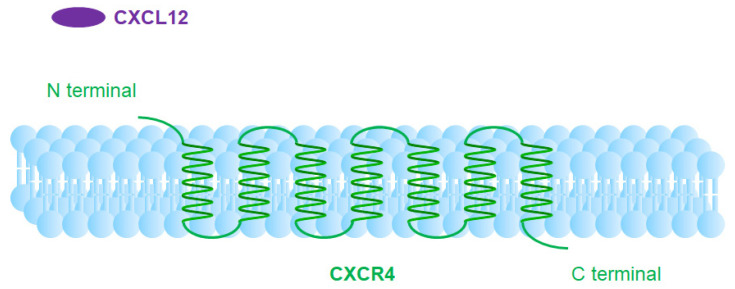
Schematic diagram of CXCR4.

The pivotal role of CXCR4 has encouraged researchers around the world to investigate CXCR4-targeted imaging and therapeutics [12]. Figure 1 illustrates the schematic diagram of CXCR4, which showed up as a 7-transmembrane helix protein. The CXCR4 crystal structure is present in the published literature [13].

## 2. Radiopharmaceuticals Based on CXCR4

The structures of CXCR4-based small molecule antagonists have been influenced by the structures of the corresponding peptide drugs to some extent. Specifically, a highly potent β-sheet-like 14-mer peptide (T140) was originally designed based on the structures of CXCR4 and CXCL12 [14]. A structure-activity relationship study of this 14-peptide showed that four of the amino acid residues (Arg2, Nal3, Tyr5, and Arg14) are indispensable for the activity of the 14-peptide.

Thus, after a series of structural optimizations, researchers obtained a pentapeptide compound, FC131, which is also a peptide compound with a high affinity for CXCR4 [15]. In this pentapeptide compound, any of the amino acid residues are very important, except for Arg2, which is less important. Taking this feature into consideration, CPCR4-2 has been further developed on the basis of FC131.

As for small molecules, a series of small molecule structures have been optimized according to the structure-activity relationship, such as pentapeptide-based antagonists, indole-based antagonists, tetrahydroquinoline-based antagonists, para-xylyl-enediamine-based antagonists, guanidine-based antagonists, quinoline-based antagonists, pyrimidine-based antagonists [13], benzenesulfonamide-based antagonists [16], etc. However, only a few of these small molecules were translated into the radiopharmaceutical field.

**Figure 2:**
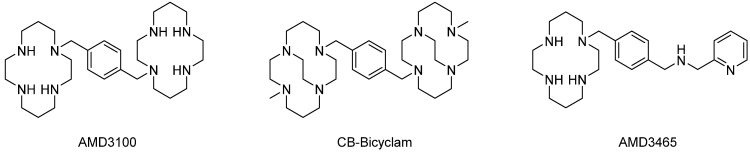
Structures of AMD3100, CB-Bicyclam, and AMD3465.

In 2009, Kiesewetter et al. [17] imply ^64^Cu-AMD3100 (Figure 2) for the first time in PET imaging of CXCR4-expressing tumors and proved that this radiotracer is useful in CXCR4-targeted imaging and therapies. They modified the synthetic route of AMD3100 in high yield and achieved ^64^Cu-AMD3100 in high radiochemical yield and with high radiochemical purity. In the binding affinity assay, ^64^Cu-AMD3100 exhibited an IC_50_ value of 62.7 μM towards Jurkat T-cells (CXCR4-positive). This value is much higher than the IC_50_ of AMD3100, verifying that the incorporation of the Cu(II) ion enhances the binding of AMD3100 to CXCR4. In the biodistribution studies, ^64^Cu-AMD3100 was observed accumulating in immune-related organs, such as the spleen (13%, 1 h post-injection), lymph nodes (10%, 1 h post-injection), and bone marrow (14%, 1 h post-injection). Other organs, such as the liver and kidney, also shared a large amount of the radiotracers [17].

In 2010, Nimmagadda et al. evaluated the kinetics and biodistribution of [^64^Cu]AMD3100 in subcutaneous brain tumor xenografts [18]. In a cell binding assay, they found [^64^Cu]AMD3100 could bind specifically to glioblastoma cell lines (U87-stb-CXCR4) and breast cancer cell lines (DU4475) with high CXCR4 expression levels. In subcutaneous tumor xenografts, this specificity was also validated [18]. In a later work in 2011, Farber et al. evaluated the extensive dosimetry in mice and established the feasibility of this radiotracer in the human body [19].

In order to bind firmly with the CXCR4 receptor, AMD3100 adopts several binding modes with the cooperation of several residues of CXCR4. One of the possible geometries is contributed by ASP171, ASP262, and GLU288, with one cyclam of AMD3100 binding to ASP171 and another cyclam binding to ASP262 and GLU288. Another possible geometry is in the form of residues ASP262 and GLU288 binding to the bicyclam rings and residues PHE189 and TYR190 binding to the methylene linker. For more binding mode information about AMD3100 and AMD3465, which is beyond the scope of this review, readers could refer to the published elegant papers [20,21,22,23]. After transition metal complex formation, the binding modes switch from electrostatic bonds between the protonated cyclam primary amine groups and aspartate residue carboxylic acid groups (ASP171 and ASP262) to coordinate bonds [24].

Except for ^64^Cu, other radioisotopes, such as ^99m^Tc, ^67^Ga, and ^62^Zn [25,26,27] were also used in the development of AMD3100-based radiopharmaceuticals, though they might possess certain shortcomings. For example, ^99m^Tc-based radiotracers are used in SPECT imaging rather than PET imaging, and [^99m^Tc]AMD3100 showed substantially reduced binding affinity toward the receptor compared with [^64^Cu]AMD3100, which might be due to the deviations in the planar structure of the cyclam caused by the introduction of a relatively large metal ion [25]; the radiolabeling time of [^67^Ga]AMD3100 is about 2 h, which is not suitable for the development of ^68^Ga-based radiotracers; besides, ^62^Zn is a seldom used isotope in labeling or imaging studies.

In 2009, Archibald et al. developed CB-Bicyclam, a cross-bridged analog of AMD3100 with a specific structure, to reinforce the interaction between the bicyclam and the aspartate residues on one surface of CXCR4 [28]. Crystal structure and computational studies confirmed the shortened and stronger interactions between the complexes and the carboxylates compared to unconstrained macrocycle complexes in AMD3100 [29]. In 2020, the same group labeled this small molecule with the radionuclide ^64^Cu to form the mono-copper species ^64^Cu-CuCB-bicyclam. Compared with AMD3100, CuCB-bicyclam reduced the up to six configurations to only one configuration when it formed the copper(II) complex. This rendered ^64^Cu-CuCB-bicyclam with higher affinity and specific binding towards CXCR4-expressing cells (U87.CXCR4 cells). Liver uptake could also be observed but could be blocked by Cu_2_CB-bicyclam [30].

In 2009, Fricker et al. investigated the pharmacology of AMD3465 [31]. In the heterologous competition binding assay, AMD3465 was used to inhibit ^125^I-SDF-1α ligand binding to CCRF-CEM cells (CXCR4 positive), with a Ki value of 41.7 ± 1.2 nM (*n* = 3). In the following assays, the authors claimed that compared with AMD3100, which possesses IC_50_ values of 651 ± 37 nM (ligand binding), 27 ± 2.2 nM (GTP binding), 572 ± 190 nM (calcium flux), and 51 ± 17 nM (chemotaxis), AMD3465 could inhibit SDF-1α-mediated cell signaling with IC_50_ values of 10.38 ± 1.99 nM (*n* = 5) (GTP binding), 12.07 ± 2.42 nM (*n* = 5) (calcium flux), and 8.7 ± 1.2 nM (*n* = 3) (SDF-1α-mediated chemotaxis). They also find that AMD3465 is a specific inhibitor of CXCR4, with an IC_50_ value against CXCR4 approximately 400-fold higher than that of AMD3465 against CCR1, CCR2b, CCR4, CCR5, CCR7, and CXCR3 [31].

In 2011, Nimmagadda et al. performed the preclinical evaluation of ^64^Cu-AMD3465 for the detection of CXCR4-expressing tumors [32]. In flow cytometric analysis, 2%, 30%, and 95% of U87 cells, HT-29 cells, and U87-stb-CXCR4 cells were CXCR4 positive. PET studies showed ID% values with the same sequence, showing this radiotracer could be used to delineate variable levels of CXCR4-expressing tumors. However, compared with AMD3100, ^64^Cu-AMD3465 exhibited superior specificity, target selectivity, and tumor-to-muscle ratios [32].

Although both [^64^Cu]AMD3465 and [^64^Cu]AMD3100 exhibited significant tumor uptake, they both showed considerable uptake in other organs, such as the kidneys, liver, and spleen. The liver uptake has been hypothesized to be partly due to transchelation of ^64^Cu, or it might be due to the relatively high lipophilicity of both compounds. It is worth mentioning that [^64^Cu]AMD3465 exhibited advanced pharmacokinetic properties compared with [^64^Cu]AMD3100, which could be attributed to the hydrophilic property of [^64^Cu]AMD3465 compared with [^64^Cu]AMD3100.

In 2018, Zhang, Lu, and Du et al. developed a ^99m^Tc-labeled antagonist based on the AMD3465 structure [33]. They evaluated the stability, binding property, and SPECT/CT performance of the complex. In a stability study, ^99m^Tc-AMD3465 could remain stable in saline and mouse serum for up to 4 h. In in vitro cellular studies, ^99m^Tc-AMD3465 exhibited radioactivity accumulation in the order CHO-CXCR4 > MCF-7 > CHO, which is in the same order as their CXCR4 expression level. In the biodistribution study, ^99m^Tc-AMD3465 showed a relative high tumor/background ratio and significant tumor uptake of 2.07 ± 0.39% ID/g, which could be blocked by AMD3465·6HBr to some extent. In SPECT/CT imaging studies, ^99m^Tc-AMD3465 exhibited a higher tumor uptake in CXCR4-positive MCF-7 tumor xenografts compared with those in CXCR4-negative CHO tumors. The uptake could be blocked by the nonradioactive species AMD3465·6HBr. However, the tumor/muscle ratio of this radiotracer was also higher in MCF-7 tumors (1.4 and 3.9 at 30 and 60 min, respectively) than in CHO tumors (1.1 and 1.5 at 30 and 60 min, respectively) [33].

In 2014, Vries et al. developed a [^11^C]Methyl-labelled CXCR4 antagonist based on AMD3465 [34]. N-[^11^C]Methyl-AMD3465 was prepared within two steps with around 60% yield, and the total synthesis time is about 50 min. In the stability assay, more than 99% of N-[^11^C]Methyl-AMD3465 remained intact after 2 h of incubation in human liver microsomes/rat plasma, showing the good stability of this radiotracer. In the binding affinity assay, N-Methyl-AMD3465 showed decreased binding affinity compared with AMD3465 but increased binding affinity compared with AMD3100. Furthermore, both biodistribution and PET studies demonstrated high and specific binding of N-[^11^C]Methyl-AMD3465 in C6 tumors, whereas the accumulation of the radiotracer in other organs, such as the liver and spleen is still high [34].

**Figure 3:**
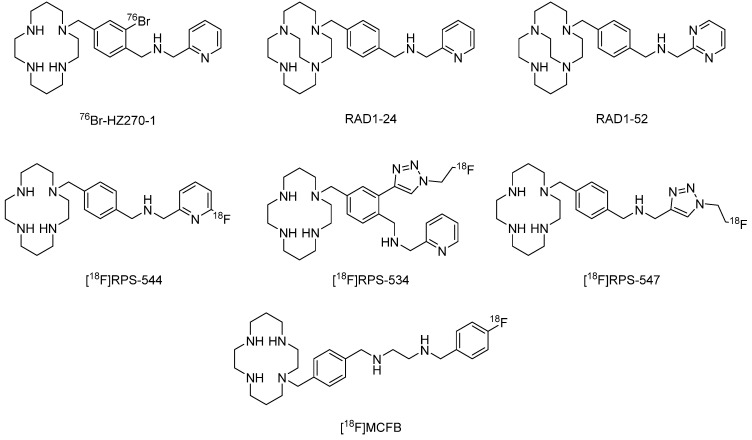
Structures of ^76^Br-HZ270-1, RAD1-24, RAD1-52, [^18^F]RPS-544, [^18^F]RPS-534, [^18^F]RPS-547, and [^18^F]MCFB.

Other radioisotopes, such as ^76^Br and ^131^I, were also labeled based on the AMD3465 and AMD3100 structures. The modification of the phenyl ring did not show an obvious change in binding affinity toward the CXCR4 binding target. Among the six studied radioligands, ^76^Br-HZ270-1 (Figure 3) exhibited the best performance for the imaging of CXCR4 expression in s.c.-located tumors rather than CNS-located tumors [35].

In 2014, Prof. Nimmagadda and his colleagues [36] developed a facile synthetic route to RAD1-24 and RAD1-52, which are cross-bridged analogs of cyclams, to evaluate their radiochemical properties. The author also tried to synthesize the side-bridged cyclam analogs. However, the trial was unsuccessful due to the instability of its exposure to oxygen peptides. In binding affinity studies, Cu(II)-coordinated compounds of RAD1-24 and RAD1-52 showed increased affinity compared to the parent cold compounds. This is largely due to the enhanced interactions between the configurationally restricted coordinated compound and the aspartate residue in the receptor binding pocket. Furthermore, high radiolabeling yields, high affinity, high tumor-to-background ratios, and prolonged target tissue residence were approved. [^64^Cu]RAD1-24 and [^64^Cu]RAD1-52 showed a higher uptake in CXCR4-positive tumors than in control tumors. The author also tried to synthesize RAD1-39, a carboxylic acid analog, in order to improve the image contrast of the cross-bridged AMD3465 analogs. However, this molecule showed neither CXCR4-specific in vitro affinity nor in vivo uptake in the tumors. This may partly be due to steric hindrance or electrostatic repulsion caused by the introduction of the carboxyl group [36].

In 2018, Babich et al. developed [^18^F]RPS-544, a [^18^F]-labeled CXCR4 antagonist based on the structure of AMD3465 [37]. [^18^F]RPS-544 is the first high affinity ^18^F-labeled CXCR4-targeted radiotracer. In a competitive binding assay, PC3-CXCR4 cells were used to evaluate the binding ability of this new tracer. As a result, the IC_50_ of RPS-544 is 4.9 ± 0.3 nM, which is located between the values of AMD3100 (50 ± 9 nM) and AMD3465 (2.7 ± 0.7 nM). In in vivo studies, [^18^F]RPS-544 displayed a tumor uptake of 3.4 ± 1.2% ID/g at 1 h post-injection in the PC3-CXCR4 tumor and 1.1 ± 0.5% ID/g in the PC3-WT tumor. However, [^18^F]RPS-544 still exhibited significant accumulation in the liver and intestines [37].

In 2019, to further improve tumor uptake and normal tissue kinetics based on [^18^F]RPS-544, Babich et al. screened more than 200 fluorine-containing structural derivatives of AMD-3465 using Schrödinger software (v.2014-3, Schrödinger, New York, NY, USA) and found that cyclam compounds containing fluoroethyltriazole groups could achieve relatively high docking scores [38]. Among these candidates, [^18^F]RPS-534 and [^18^F]RPS-547 exhibited superior properties compared with [^18^F]RPS-544. To be specific, tumor uptake of [^18^F]RPS-547 (3.09 ± 0.52% ID/g) was comparable to [^18^F]RPS-544 (3.4 ± 1.2% ID/g), and the rapid clearance of [^18^F]RPS-547 led to higher tumor/background ratios. When it comes to [^18^F]RPS-534, the tumor uptake (7.2 ± 0.3% ID/g) and tumor/background ratios were even greater than [^18^F]RPS-544 and comparable to [^68^Ga]Pentixafor [38].

In 2019, Aboagye et al. developed a radiotracer named [^18^F]MCFB based on the structure of AMD3465 due to its superior binding affinity and selectivity toward CXCR4 [39]. To avoid defluorination of the 2-fluoropyridine and 4-fluoropyridine, fluorobenzene was taken into consideration as a design strategy. The logD_octanol/PBS_ of [^18^F]MCFB was −1.64 ± 0.06, which was moderate among the other reported radiotracers. In the binding assay, [^19^F]MCFB showed an IC_50_ value of 111.3 nM, which is comparable to that of AMD3465 (89.8 nM). In in vitro binding studies, both U2932 (higher CXCR4 expression) and SuDHL8 (lower CXCR4 expression) cell lines were used. The uptake of [^18^F]MCFB in the U2932 cell line was higher than in the SuDHL8 cell line. However, the addition of AMD3465 will lead to partial inhibition of the binding, which means the existence of partial nonspecific binding. Even though the specific uptake was sensitive to the CXCR4 expression level, knockdown of CXCR4 in the MDA-MB-231 shCXCR4 cell line with shRNA decreased the [^18^F]MCFB uptake, which is consistent with the decrease in the CXCR4 expression level. In PET studies, [^18^F]MCFB showed almost two-fold higher uptake in the U2932 tumor than in the SuDHL8 tumor, which is consistent with the CXCR4 expression level. In biodistribution studies, tumor uptake in U2932 was still higher than SuDHL8. However, bone uptake was relatively low, showing a low possibility of defluorination [39].

**Figure 4:**
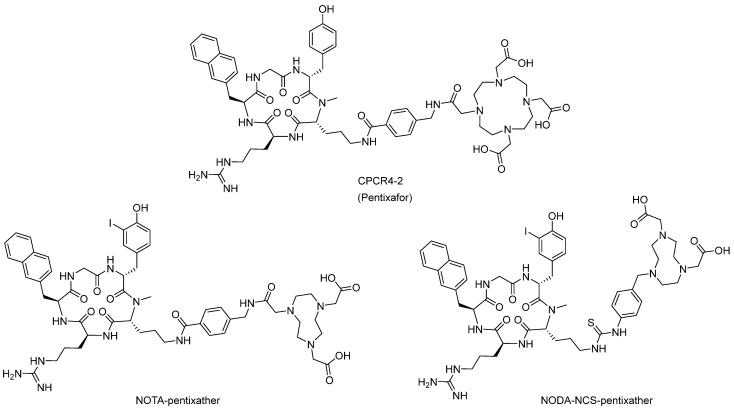
Structures of CPCR4-2 (Pentixafor), NOTA-pentixather, and NODA-NCS-pentixather.

The first ^68^Ga-labeled CXCR4 imaging probe was published by Wester and his collaborators. In 2011, Wester et al. developed CPCR4-2 (Pentixafor), a small cyclic pentapeptide-based molecule, to chelate with ^68^Ga^3+^ through the interaction with the DOTA moiety (Figure 4) [40]. In the binding assay, the indium complex of CPCR4-2 exhibited an affinity of 44 ± 4 nM towards Jurkat cells (CXCR4-positive), while the binding affinities of the gallium complex were 5 ± 1 nM, which is comparable to the unmodified pentapeptide FC131. In in vivo studies carried out in nude mice bearing human small cell lung cancer tumor xenografts, ^68^Ga^3+^-labeled CPCR4-2 showed CXCR4-specific tumor uptake, fast renal excretion, and high tumor-to-muscle ratios.

In 2011, the same group carried out further pharmacologic studies of CPCR4-2. In the lipophilicity assay, ^68^Ga-CPCR4-2 (^68^Ga-Pentixafor) displayed enhanced hydrophilicity with a logP_octanol/PBS_ of 22.90 ± 0.08, which is much higher than that of ^125^I-FC131. Competition binding studies and biodistribution studies showed that ^68^Ga-CPCR4-2 possesses high and specific tumor accumulation and low uptake in the nontumor region, leading to high-contrast images of tumors in small-animal PET studies [41].

In order to evaluate the PET imaging property of ^68^Ga-Pentixafor in patients with solid tumors, Vag et al. (2016) performed PET imaging experiments on 21 patients with histologically proven pancreatic cancer, laryngeal cancer, non-small cell lung cancer (NSCLC), prostate cancer, etc. [42]. Moreover, ^18^F-FDG was also used in 10 out of 21 patients with a total of 27 lesions as a comparison. This comparison between ^68^Ga-Pentixafor and ^18^F-FDG demonstrates that ^18^F-FDG is superior to ^68^Ga-Pentixafor. For example, among the 27 lesions evaluated, only 19 of the 27 lesions could be detected with ^68^Ga-Pentixafor, whereas ^18^F-FDG could detect all 27 lesions. However, among all measured lesions, ^18^F-FDG demonstrated significantly higher SUV_max_ and T/B ratios compared with ^68^Ga-Pentixafor [42].

In 2019, Li et al. further evaluated the performance of ^68^Ga-Pentixafor and ^18^F-FDG PET/CT in newly diagnosed multiple myeloma [43]. 30 patients were enrolled, and among them, ^68^Ga-Pentixafor (28/30, 93.3%) showed a higher positive rate than ^18^F-FDG (16/30, 53.3%) in the PET/CT study. In ^68^Ga-Pentixafor PET/CT, 18 patients showed intense radioactivity uptake (SUV_max_ of 17.0 ± 15.1) in the bone marrow, and 10 patients showed moderate uptake (SUV_max_ of 5.8 ± 1.3). In ^18^F-FDG PET/CT, only 15 patients showed moderate bone marrow uptake (SUV_max_ of 7.1 ± 4.9) [43].

In addition, ^68^Ga-pentixafor has also been studied by researchers in other types of tumors, such as multiple myeloma [44,45,46,47,48], leukemia [49,50], adrenocortical carcinoma [51], glioblastoma [52], small-cell lung cancer [53,54], non-small-cell lung cancer [55], lymphoproliferative diseases [56], neuroendocrine tumors [57,58], extranodal marginal zone lymphoma (a subtype of non-Hodgkin’s lymphoma) [59], and esophageal adenocarcinoma [60]. Readers who are interested could refer to this literature.

Based on the structure of pentixafor, Poschenrieder et al. [61] developed the other two pentapeptide-based structures, NOTA-pentixather and NODA-NCS-pentixather. Both molecules were synthesized and labeled with Al[^nat^F], while only [^18^F]AlF-NOTA-pentixather was evaluated in vitro and in vivo. The logP_octanol/water_ value of [^18^F]AlF-NOTA-pentixather was −1.4, which is a little higher than [^68^Ga]pentixafor (−2.9). This could be due to the one less carboxylate group in NOTA compared with DOTA. As a result, [^18^F]AlF-NOTA-pentixather showed increased accumulation in the gall bladder and intestines. In the binding affinity assay, [^nat^F]AlF-NOTA-pentixather showed 1.4-fold higher CXCR4 affinity compared with [^nat^Ga]pentixafor. Both [^18^F]AlF-NOTA-pentixather and [^68^Ga]pentixafor were evaluated in a biodistribution study conducted in Daudi xenograft-bearing mice. In accordance with the hydrophilic trend, [^18^F]AlF-NOTA-pentixather showed delayed blood clearance. Furthermore, relatively high bone activity levels were observed, which was attributed to the defluorination phenomenon. However, both high CXCR4-specific in vivo uptake and high contrast in PET imaging were observed, and thus this molecule once again demonstrates the excellent properties of pentapeptide (FC131)-based radiotracers [61].

**Figure 5:**
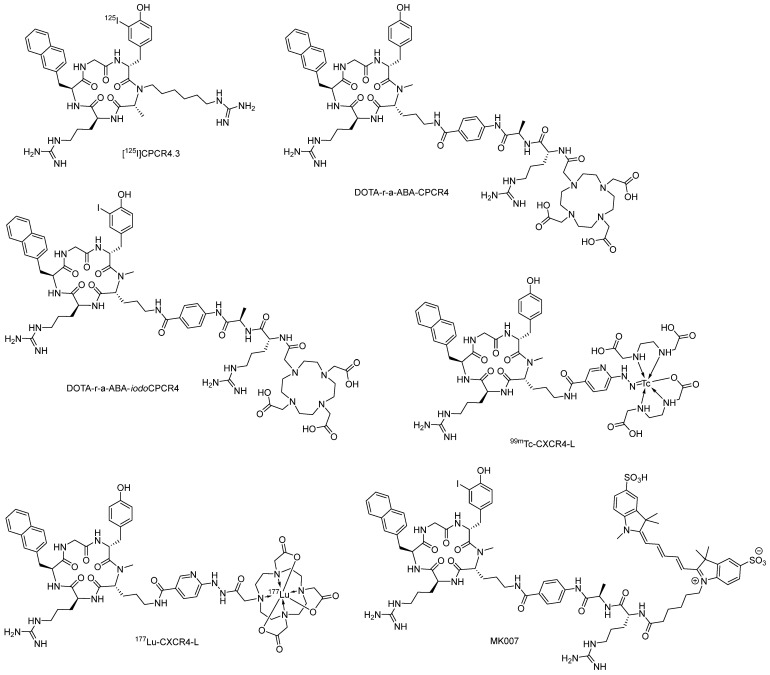
Structures of [^125^I]CPCR4.3, DOTA-r-a-ABA-CPCR4, DOTA-r-a-ABA-*iodo*CPCR4, ^99m^Tc-CXCR4-L, ^177^Lu-CXCR4-L, and MK007.

[^68^Ga]Pentixafor ([^68^Ga]CPCR4-2) holds its selectivity only for human chemokine receptor 4 (hCXCR4) instead of murine chemokine receptor 4 (mCXCR4). To solve this problem, Schottelius et al. [62] developed [^125^I]CPCR4.3 (Figure 5) based on the structure of CPCR4-2, which could be used in in vitro and in vivo targeting of hCXCR4 and mCXCR4. In human cancer cell lines with different endogenous hCXCR4 expression levels, such as Jurkat, Daudi, HT-19, MCF-7, SH-5YSY, and LNCaP, [^125^I]CPCR4.3 exhibited 2.4 to 11 fold increased binding compared with [^68^Ga]Pentixafor. Furthermore, in cancer cell lines with different mCXCR4 expression levels, such as Eμ-myc 1080 and 4 T1, [^125^I]CPCR4.3 showed strong and specific binding. In comparison, [^68^Ga]Pentixafor showed virtually no binding to mCXCR4. Taking the biodistribution study into consideration, [^125^I]CPCR4.3 still holds promise in the development of preclinical models expressing mCXCR4, even though high accumulation levels could be observed in the liver and intestine [62].

With the development of [^68^Ga]Pentixafor and [^68^Ga]Pentixather and their clinical achievements, researchers tend to modify the fine chemical structures of these two tracers to further improve the ligand-receptor interaction. In 2020, Schottelius et al. [63] replaced the AMBA-linker in [^68^Ga]Pentixafor and [^68^Ga]Pentixather with a series of new linkers. After scrutinizing affinity data and cellular uptake studies among these new tracers labeled with [^nat^Ga], [^nat^Lu], [^nat^Y], and [^nat^Bi], DOTA-r-a-ABA-CPCR4 and DOTA-r-a-ABA-*iodo*CPCR4, labeled with [^68^Ga] for PET imaging and [^177^Lu] for therapeutic application, were selected for further evaluation. In the internalization study, both [^177^Lu]DOTA-r-a-ABA-CPCR4 and [^177^Lu]DOTA-r-a-ABA-*iodo*CPCR4 showed more than 4-fold total cellular uptake than [^177^Lu]Pentixather, which is identical with their affinity results. In the lipophilicity assay, due to the cationic nature of the r-a-ABA structure, the introduction of the r-a-ABA linker led to a generally reduced lipophilicity of [^68^Ga/^177^Lu]DOTA-r-a-ABA-CPCR4 and [^68^Ga/^177^Lu]DOTA-r-a-ABA-*iodo*CPCR4 compared with the reference ligands [^68^Ga]Pentixafor and [^177^Lu]Pentixather. In the biodistribution study, [^177^Lu]DOTA-r-a-ABA-CPCR4 and [^177^Lu]DOTA-r-a-ABA-*iodo*CPCR4 exhibited superior tumor accumulation and retention up to 48 h post-injection compared with the reference tracer [^177^Lu]Pentixather, which is consistent with their affinity trend and cellular uptake properties. However, in the PET study, both [^68^Ga]DOTA-r-a-ABA-CPCR4 and [^68^Ga]DOTA-r-a-ABA-*iodo*CPCR4 showed inferior imaging quality compared with [^68^Ga]Pentixafor, which might be due to the enhanced background signal [63].

In 2020, Ferro-Flores and Jiménez-Mancilla et al. [64] developed ^99m^Tc and ^177^Lu labeled pentapeptide-based CXCR4 targeted radiotracer pairs, ^99m^Tc-CXCR4-L and ^177^Lu-CXCR4-L, for theranostic purposes. In molecular docking calculations, the theoretical affinity of HYNIC-CXCR4-L (−10.9 kcal/mol) was comparable with that of CVX15 (−9.2 kcal/mol, cyclopeptide co-crystallized with CXCR4 monomer downloaded from the RCSB Protein Data Bank). In the study in cancer cells, the uptake of ^177^Lu-CXCR4-L in DU-4475 and C6 cells (CXCR4-positive) was significantly higher than that of ^99m^Tc-CXCR4-L in the same cells. However, in the aspect of internalization, ^177^Lu-CXCR4-L showed a lower value than ^99m^Tc-CXCR4-L, which might be due to the chemical effect of the DOTA structure in ^177^Lu-CXCR4-L. In biodistribution studies, ^99m^Tc-CXCR4-L showed 2.5% ID/g after 3 h post-injection, whereas ^177^Lu-CXCR4-L showed 1.5% ID/g after 96 h post-injection. Micro-SPECT/CT images of the same animal injected with ^99m^Tc-CXCR4-L at day 0 and ^177^Lu-CXCR4-L at day 2 clearly showed the tracer uptake in the CU-4475 and the C6 tumors, demonstrating the possibility of this radiotracer pair as a theranostic pair [64]. Further, nine patients with evidence (MRI) of brain tumors were screened with SPECT after ^99m^Tc-CXCR4-L injection, and seven of them were diagnosed as grade II oligodendroglioma, grade IV glioblastoma, grade IV gliosarcoma, metastasis, or diffuse astrocytoma. The other two negative SPECT patients were diagnosed with reactive gliosis, confirmed with immunohistochemistry [65].

Due to its superior binding property toward CXCR4, the pentapeptide moiety (CPCR4) was introduced into other imaging methods, such as NIR (near-infrared) fluorescence imaging. In 2022, Quante et al. [66] developed MK007, in which the CPCR4 structure was conjugated to the sulfo-Cy5 moiety with the help of a linker. The lipophilicity was determined to be −1.83 ± 0.02 using [^125^I]MK007. This new fluorescence probe was determined to be a superior probe for NIR fluorescence imaging [66].

**Figure 6:**
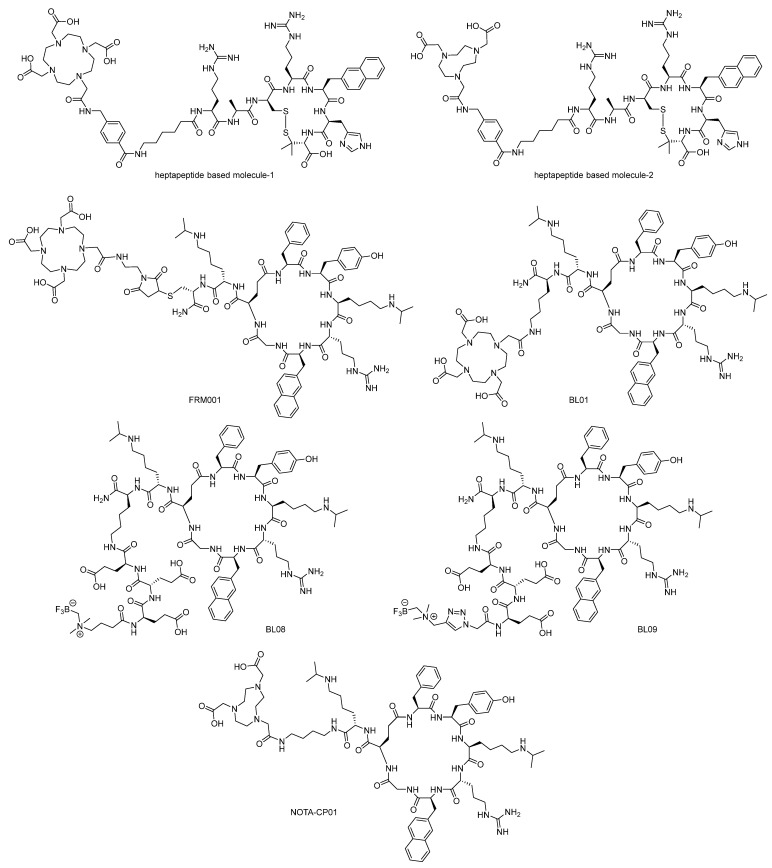
Structures of heptapeptide-based molecule-1, heptapeptide-based molecule-2, FRM001, BL01, BL08, BL09, and NOTA-CP01.

In 2021, Scala and Lastoria et al. [67] developed another two cyclic peptide radiotracers ([^68^Ga]-4 and [^68^Ga]-5, herein referred to as [^68^Ga]heptapeptide-based molecule-1 and [^68^Ga]heptapeptide-based molecule-2, Figure 6) based on a previously constructed heptapeptide structure. This heptapeptide structure is derived from CXCL12 through a series of modifications. In binding assays, heptapeptide-based molecule-1 (203 ± 78 nM) and heptapeptide-based molecule-2 (42 ± 19 nM) exhibited decreased affinity compared with their parent peptide (5.1 ± 3.8 nM), due to the introduction of bulky structures DOTA and NOTA. However, formation of complexes with [^nat^Ga] rendered them with compensation of affinity (49 ± 15 nM for [^nat^Ga]heptapeptide-based molecule-1 and 15.6 ± 4.2 nM for [^nat^Ga]heptapeptide-based molecule-2). In the lipophilicity study, [^nat^Ga]heptapeptide-based molecule-1 and [^nat^Ga]heptapeptide-based molecule-2 showed similar partition coefficient data (−1.51 vs. −1.60). Biodistribution studies and PET imaging studies were performed in CHO-hCXCR4-bearing CD1 mice and Daudi lymphoma-bearing SCID mice. In CHO-hCXCR4-bearing CD1 mice, both [^68^Ga]heptapeptide-based molecule-1 and [^68^Ga]heptapeptide-based molecule-2 exhibited rapid clearance and low accumulation levels in background tissues. Compared with [^68^Ga]heptapeptide-based molecule-1, [^68^Ga]heptapeptide-based molecule-2 showed a higher specific accumulation level in the tumor region and a higher tumor/background ratio, which is consistent with the affinity trend [67].

LY2510924 is another cyclic peptide structure suitable for CXCR4 targeting and with potent antitumor activities [68]. In 2019, Suzuki et al. [69] developed FRM001 based on the structure of LY2510924 conjugated with the DOTA (1,4,7,10-tetraazacyclododecane-1,4,7,10-tetraacetic acid) moiety. In the binding affinity assay, the IC_50_ value of FRM001 (1.78 ± 0.15 nM) was similar to that of its parent peptide, LY2510924 (1.37 ± 0.10 nM). Complexation with Ga^3+^, Lu^3+^, and Y^3+^ did not change the IC_50_ value of FRM001 too much. As a comparison, FC131, AMD3465, and AMD3100 showed almost 10 times higher IC_50_ values. In the internalization assay, FRM001 was labeled with ^67^Ga to determine the internalization activity, and the majority of [^67^Ga]FRM001 was found to remain at the cell membrane rather than be internalized. A biodistribution study of [^67^Ga]FRM001 was performed in CCRF-CEM tumor-bearing mice, and [^67^Ga]FRM001 showed a high tumor-to-blood ratio of 59 at 4 h post-injection. Similar to other CXCR4-targeted radiotracers, the hepatic accumulation was still high to some extent. However, co-injection with AMD3100 will reduce this accumulation. A similar phenomenon was observed in the PET image acquired at 1 h post-injection [69].

**Figure 7:**
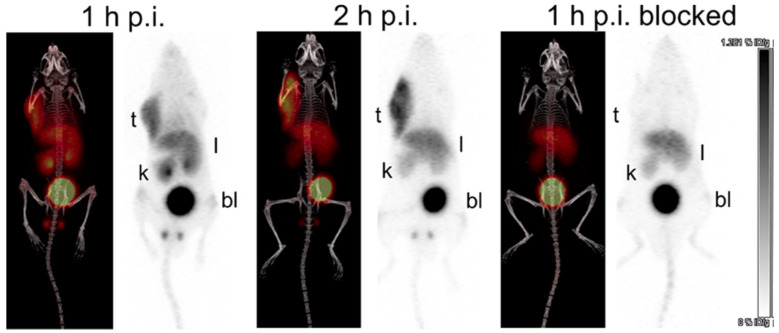
PET/CT and PET alone maximal intensity projections of [^68^Ga]Ga-BL01 at 1 and 2 h postinjection in mice bearing Daudi Burkitt’s lymphoma xenografts. The blocking study was performed by injection of LY2510924 15 min before tracer administration. The scale bar is in units of %ID/g from 0 to 1.2 × 10 (t = tumor; l = liver; k = kidney; bl = bladder). Reprinted with permission from [70]. Copyright © 2019 American Chemical Society.

As LY2510924 is a potent peptide antagonist of CXCR4, in 2019, Bénard and Lin et al. [70] developed BL01 based on the structure of this cyclic nonapeptide. Radionuclides ^68^Ga and ^177^Lu were used to form complexes with BL01, and assays such as affinity and biodistribution were conducted on the tracers. In binding affinity assays, the IC_50_ values of LY2510924, Ga-BL01, and Lu-BL01 are 27.8 ± 7.4, 21.2 ± 15.9, and 7.1 ± 1.7 nM, respectively. These data demonstrate that the introduction of the DOTA moiety and complex formation, especially the Lu^3+^-based complex formation, contribute to the increased binding affinity. The logD values of Ga-BL01 and Li-BL01 are −3.36 ± 0.09 and −3.34 ± 0.10, respectively, demonstrating the dominant renal clearance pathway. In the PET/CT study, [^68^Ga]Ga-BL01 uptake could be observed in tumors, the liver, kidneys, and bladder. The tumor region could be clearly observed, and preinjection of LY2510924 could significantly reduce tumor uptake (Figure 7). In a biodistribution study, preinjection of LY2510924 could significantly reduce the uptake of [^68^Ga]Ga-BL01 and [^177^Lu]Lu-BL01 in tumors. For [^177^Lu]Lu-BL01 in 1 h post-injection, tissue uptake was 12.95 ± 1.27% ID/g (lung), 11.55 ± 1.78% ID/g (spleen), and 14.00 ± 1.12% ID/g (tumor), respectively. At 4 h post-injection, the tumor-to-blood and tumor-to-muscle ratios of [^177^Lu]Lu-BL01 were 92.9 ± 24.7 and 105 ± 24.8, respectively. At 24 h post-injection, these ratios increased to 229 ± 32.9 and 131 ± 27.7, respectively. At 24 h and 72 h post-injection, tumor uptake of [^177^Lu]Lu-BL01 was 10.09 ± 1.41 and 3.62 ± 0.68% ID/g, respectively [70].

**Figure 8:**
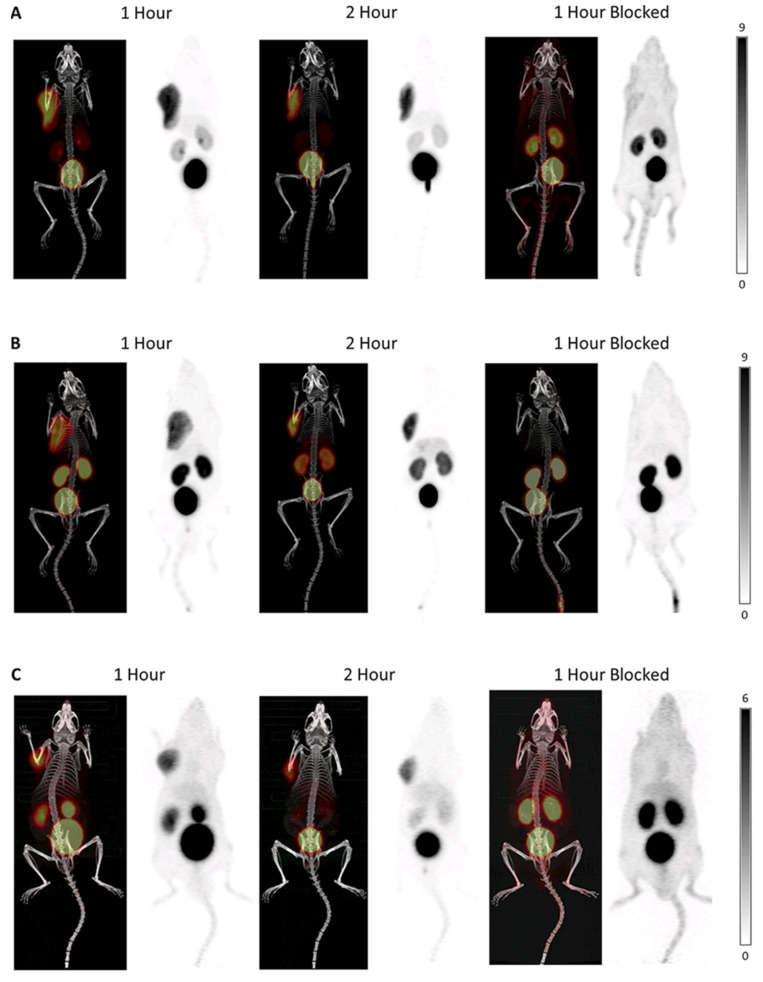
Maximum intensity projections for PET/CT and PET alone at 1 h p.i., 2 h p.i., and 1 h p.i., blocking (**A**) [^18^F]BL08, (**B**) [^18^F]BL09, and (**C**) [^68^Ga]Ga-Pentixafor. The blocking was performed via injection of LY2510924 15 min prior. The scales of the PET images of [^18^F]BL08 and [^18^F]BL09 are 0–9% ID/g, and the scale of the PET images of [^68^Ga]Ga-Pentixafor is 0–6% ID/g. Reprinted with permission from [71]. Copyright © 2021 American Chemical Society.

However, [^68^Ga]Ga-BL01 and [^177^Lu]Lu-BL01 showed relatively high peripheral tissue accumulation. In such a scenario, the author continued to develop LY2510924-based antagonists and achieved ^18^F-labeled radiotracers [^18^F]BL08 and [^18^F]BL09. In binding affinity assays, [^18^F]BL08 (11.6 ± 7.0 nM) and [^18^F]BL09 (13.4 ± 2.3 nM) showed much improved binding affinity compared with [^68^Ga]Ga-Pentixafor (24.8 ± 2.5 nM). The partition coefficients of these two radiotracers are comparable to those of [^68^Ga]Ga-Pentixafor (−2.90), and among these two tracers, [^18^F]BL08 (−3.45 ± 0.33) was more hydrophilic than [^18^F]BL09 (−2.49 ± 0.19). PET/CT and biodistribution studies showed that both [^18^F]BL08 and [^18^F]BL09 exhibited specific uptake in Daudi xenograft-bearing mice ([^18^F]BL08: 7.60 ± 1.38% ID/g, [^18^F]BL09: 6.61 ± 2.07% ID/g). In blocking experiments (Figure 8), the tumor uptake data decreased to 1.17 ± 0.71% ID/g ([^18^F]BL08) and 0.79 ± 0.65% ID/g ([^18^F]BL09). Furthermore, both radiotracers showed little uptake in the peripheral organs. For example, tumor/muscle ratios at 2 h are 339.0 ± 81.4 for [^18^F]BL08 and 238.6 ± 72.0 for [^18^F]BL09, while in the previous work the same ratio was 52.87 ± 2.26 for [^68^Ga]Ga-BL01 at the 2 h timepoint. Moreover, compared with [^18^F]BL08 and [^18^F]BL09, [^68^Ga]Ga-Pentixafor exhibited a relatively lower tumor/background ratio [71].

In 2021, Jin et al. [72] developed an LY2510924-based radiopharmaceutical, [^64^Cu]NOTA-CP01, which is conjugated with the NOTA moiety and labeled with ^64^Cu. [^64^Cu]NOTA-CP01 was stable in saline and FBS within 12 h of incubation. In partition coefficient evaluation, [^64^Cu]NOTA-CP01 showed a logP value of −3.44 ± 0.12, which means a relatively high hydrophilic property. Competitive binding studies showed that the binding of [^64^Cu]NOTA-CP01 to CXCR4 was specific, and the calculated IC_50_ value was 1.61 ± 0.96 nM. In a micro-PET/CT imaging study and biodistribution study, [^64^Cu]NOTA-CP01 was injected into EC109 tumor-bearing mice (CXCR4-positive). Among the PET images captured during 0.5–24 h, the image at 6 h was the best due to the uptake of [^64^Cu]NOTA-CP01 in the EC109 tumor. At the 6 h timepoint, the tumor/blood and tumor/muscle ratios of the radiotracer are 4.79 ± 0.06 and 15.44 ± 2.94, respectively. However, the liver uptake of [^64^Cu]NOTA-CP01 was still high, though the blood clearance was fast due to the hydrophilic property of the molecule [72].

**Figure 9:**
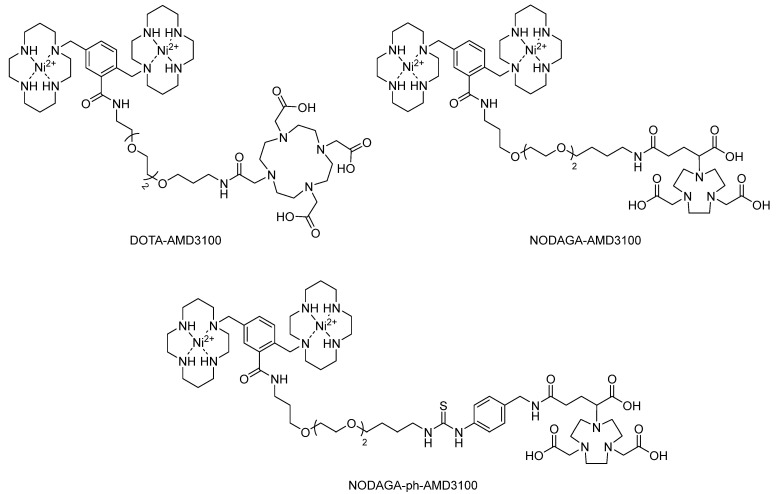
Structures of DOTA-AMD3100, NODAGA-AMD3100, and NODAGA-ph-AMD3100.

In 2016, to overcome the high liver uptake of ^64^Cu-AMD3100, Denat et al. [73] developed three radiotracers, AMD3100-DOTA, AMD3100-NODAGA, and AMD3100-ph-NODAGA, based on the AMD3100 moiety and DOTA/NODAGA chelators, using PEG_3_ as a linker (Figure 9). The PEG_3_ linker was introduced to reduce the high lipophilicity of ^64^Cu-AMD3100, and the DOTA/NODAGA moiety was introduced to avoid the metal release phenomenon. However, to avoid radiolabeling of cyclam with ^68^Ga, Ni^2+^ ions were used as blocking reagents because the Ni^2+^/cyclam complex was stable even in strong acidic solutions and the only method to remove Ni^2+^ ions from cyclam was to form cyanide at high temperature.

The IC_50_ values of ^nat^Ga-AMD3100-DOTA, ^nat^Ga-AMD3100-NODAGA, and ^nat^Ga-AMD3100-ph-NODAGA are 516, 1485, and 121 nM, respectively. The affinities of these complexes are all lower than those of AMD3100, which are 14 nM. A flow cytometry assay confirmed this tendency. Binding and internalization assays demonstrated that ^68^Ga-AMD3100-ph-NODAGA possesses higher total cell uptake (1.77 ± 0.10%) compared with ^68^Ga-AMD3100-DOTA (0.61 ± 0.22%), indicating that ^68^Ga-AMD3100-ph-NODAGA might be suitable for the biodistribution study and PET imaging. Unfortunately, in H69 xenograft (CXCR4 positive) bearing nude mice, ^68^Ga-AMD3100-ph-NODAGA showed lower accumulation of the radioactivity in the tumor than ^64^Cu-AMD3100, with comparable accumulation in immune-related organs [73].

In 2016, Blasberg et al. developed an ^18^F-labeled pyrimidine-pyridine amine as a radiotracer for the detection of CXCR4 receptors in gliomas ([^18^F]RPS-510). It belongs to a para-xylyl-enediamine-based structure. However, it showed no specific binding toward CXCR4-overexpressing U87 cells [74]. Another para-xylyl-enediamine-based radiotracer named [^18^F]MSX-122F was developed by Shim et al. in 2012 [75]. However, there was no in vivo data collected by the authors [75].

**Figure 10:**
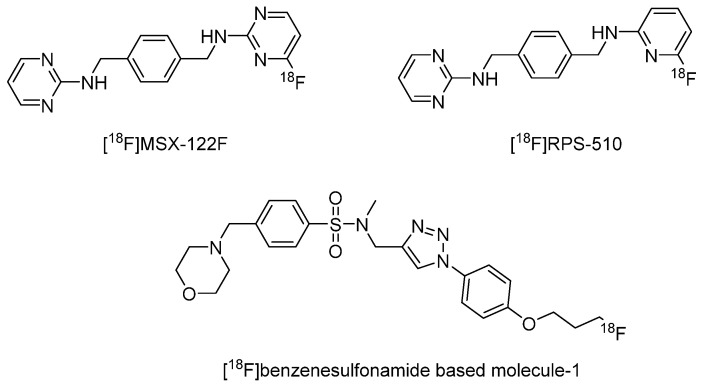
Structures of [^18^F]RPS-510, [^18^F]MSX-122F, and [^18^F]benzenesulfonamide-based molecule-1.

In 2020, Shim et al. [76] developed a benzenesulfonamide-based radiotracer (compound 5, herein referred to as [^18^F]benzenesulfonamide-based molecule-1) according to the optimizing result of the Schrödinger Suite. The optimization process has considered each atom’s contribution to the entire benzenesulfonamide-based molecule developed by the author previously. As a result, the negatively contributing moiety was replaced to introduce the radionuclide F-18. The formed [^18^F]benzenesulfonamide-based molecule-1 is shown in Figure 10. In an in vitro binding assay, it showed an IC_50_ of 6.9 nM to block TN14003, which is a CXCR4-targeted peptide. At the same condition, AMD3100 showed an IC_50_ of 66 nM. In in vivo imaging studies, [^18^F]benzenesulfonamide-based molecule-1 showed significantly higher radioactivity in the lesion of paw edema, which demonstrates its ability to visualize γ-carrageenan-induced inflammation. Furthermore, [^18^F]benzenesulfonamide-based molecule-1 exhibited preferential accumulation in the lesion of orthotopic xenograft SCCHN (4.00 ± 0.28% ID/g) and metastatic tumors arising in the lung (1.66 ± 0.14% ID/g), though the uptake in bone marrow was also specific [76].

## 3. Current Clinical and Marketing Information [77]

At present, there are about 76 potential small molecular and peptide drugs (including chemical drugs and radiopharmaceuticals) targeting the CXCR4 preclinical and clinical stages, but only one drug (AMD3100, Plerixafor, MOZOBIL) has been approved for marketing. The number of related drugs filed in China is about 36. In the field of CXCR4, there are about 200 clinical studies in progress worldwide, of which less than 10 are in China.

In the aspect of chemical drugs, the small molecular drugs entering clinical trials on the Chinese mainland are those based on the structure of AMD3100 (Plerixafor), which is generally used for the treatment of non-Hodgkin’s lymphoma or multiple myeloma. Phase III clinical trials of AMD3100 have been completed on the Chinese mainland (registration date: July 2014, for the treatment of non-Hodgkin’s lymphoma), sponsored by Genzyme Corporation (Cambridge, MA, USA), Patheon UK Ltd. (Wiltshire, UK), and Labcorp drug development (Beijing, China) Co., Ltd., at the Peking University People’s Hospital. While the study for the treatment of multiple myeloma with AMD3100 has entered Phase IV in China at the First Affiliated Hospital of Soochow University, with a registration date of July 2021.

Internationally, small molecules and peptides that entered clinical trials were also dominated by the structure of AMD3100 (Plerixafor) in the US, EU (European Union), Australia, Japan, and many other countries and regions.

In addition, a large number of other drugs, such as AMD3465, TN14003, and GSK812397, are also in preclinical studies. LY2510924 is in clinical phase I for metastatic pancreatic cancer, metastatic rectal cancer, and advanced solid tumors; Burixafor is in clinical phase II for the treatment of non-Hodgkin’s lymphoma, multiple myeloma, acute myeloid leukemia, Hodgkin’s disease, haematological neoplasms, etc.; Motixafortide is in registration period for the treatment of multiple myeloma, haematological neoplasms, etc.; Balixafortide is in clinical phase III for the treatment of recurrent metastatic breast cancer, multiple myeloma, metastatic breast cancer, acute myocardial infarction, myocardial infarction, HIV infection, acute myeloid leukaemia, HER2-negative breast cancer, etc.

Small molecules and peptides targeting CXCR4 are currently limited to Plerixafor injection in international markets, which was first marketed in the US in 2008, then in the EU in 2009, in Canada in 2012, and in China in recent years (2019–2022), with companies, such as Sichuan Huiyu Pharmaceutical Co., Ltd. (Neijiang, China), Hefei Yifan Biopharmaceutical Co., Ltd. (Hefei, China), and Hunan Wuzhoutong Pharmaceutical Co., Ltd. (Xiangtan, China) being approved to produce it.

Compared with the field of chemical drugs, in the field of radiopharmaceuticals, there are few types of drugs that have reached the clinical trial stage.

Radiopharmaceuticals that target CXCR4 are mainly ^68^Ga-Pentixafor, which has entered Phase II clinical trials in the US and EU with PentixaPharm GmbH as the sponsor and has entered Phase I clinical trials in China with several sponsors, including First Affiliated Hospital of Fujian Medical University, Peking Union Medical College Hospital, and Zhongnan Hospital of Wuhan University. Furthermore, this drug has also entered the clinical phase in Australia, with Royal Brisbane and Women’s Hospital as the sponsor. The drug is mainly used for PET imaging and can be used to diagnose hematological tumors, secondary CNS lymphomas, multiple myeloma, lymphomas, primary CNS lymphomas, etc.

In addition, ^68^Ga-Pentixather, ^212^Pb-Pentixather, ^177^Lu-Pentixather, and ^90^Y-Pentixather are also in the clinical phase.

There are currently no drugs targeting CXCR4, either internationally or on the Chinese mainland, that are available on the market in the radiopharmaceutical field.

MOZOBIL’s worldwide sales figures show an annual average sale of approximately 200 million euros over the last five years, with year-on-year growth [77].

Considering the paramount role of CXCR4 in the human body, we anticipate that with the development of CXCR4-targeting radiopharmaceuticals in preclinical research and clinical trials, CXCR4-targeting radiopharmaceuticals will have a prosperous future.

## 4. Perspective Development

CXCR4-based radiopharmaceuticals are likely to have two main development directions in the future:

On the one hand, compared to CXCR4-based chemotherapeutics, CXCR4-based radiopharmaceuticals are currently receiving attention within only a few molecular structures, e.g., AMD3100, Pentixafor, Pentixather, etc. In recent years, LY2510924, a peptide that was radionuclide-labeled, has also undergone certain preclinical studies. However, there are still a large number of chemical drug structures that have reached the clinical study stage but have not been radionuclide-labeled for further adequate study, and there are also still potential molecular structures that have not yet been designed and molecular docking simulated. The successful translation of these potential radiopharmaceutical precursors will not only enable a more comprehensive range of CXCR4-based radiopharmaceuticals, which will help the drugs be effective against a wider range of cancer types, but will also hopefully address the shortcomings of existing CXCR4-based radiopharmaceuticals with high hepatotoxicity. Therefore, radiopharmaceutical practitioners should not only pay attention to the progress of the development and marketing of popular radiopharmaceutical precursors, such as AMD3100 and Pentixafor, for generic drug development but also pay attention to and try to develop other novel structures for innovative drug research.

On the other hand, the rise of multimodal technologies has made it possible to combine the technical means of PET and SPECT, on which radiopharmaceuticals are based, with other technical means, such as photothermal therapy and chemotherapy [66,78,79]. Such research has helped to deepen our understanding of the underlying mechanisms and intermolecular interactions and has also helped to promote the creation of new instruments and assays. However, such technologies are currently largely confined to universities and research institutes, with less attention paid to them by for-profit companies.

The application prospects of CXCR4-targeted radionuclide therapy are very promising. According to the existing literature, in the early detection of multiple myeloma, the radiotracer ^68^Ga-Pentixafor showed a higher positive rate than the commonly used radiopharmaceutical ^18^F-FDG. In addition, in the aspect of radionuclide labeling, it is difficult to modify the structure of ^18^F-FDG to realize the application of radiotherapy. In contrast, ^68^Ga-Pentixafor could realize radiotherapy through structural modification (^177^Lu-Pentixather). On the other hand, in terms of metabolism in the body, polypeptide drugs are more beneficial to radiotherapy than small molecules such as ^18^F-FDG. In general, CXCR4-targeted radiopharmaceutical research is conducive to the development of integrated diagnosis and treatment. In addition, CXCR4 is not limited to the diagnosis and treatment of multiple myeloma. Since it is overexpressed on the surface of more than 23 types of human cancer cells, including non-Hodgkin lymphoma, multiple myeloma, chronic lymphocytic leukemia, and acute myeloid leukemia, it will also make progress in the detection and treatment of other types of tumors.

## 5. Conclusions

Overall, the development of CXCR4-based radiopharmaceuticals is still on the rise and requires the concerted efforts of researchers around the world, as well as the collaboration of staff from companies, universities, and research institutes.

## Data Availability

No new data were created.

## Sample Availability

Not available.
